# Economic value of high-energy, high-protein oral nutritional supplements in disease-related malnutrition in the French community setting

**DOI:** 10.3389/fnut.2026.1952501

**Published:** 2026-10-14

**Authors:** Valérie Carot, Aditya Dular, Marie Le Novere, Laetitia Schmitt

**Affiliations:** 1 Nutricia Nutrition Clinique, Rueil-Malmaison, France; 2 Nutricia Global, Hoofddorp, Netherlands; 3 Clarivate Analytics, London, United Kingdom

**Keywords:** community, cost savings, disease-related malnutrition, frailty/sarcopenia, France, oral nutritional supplements

## Abstract

**Objectives:**

Despite growing evidence supporting nutritional interventions, the comparative economic value of the highest high-energy, high-protein (H-HEHP) oral nutritional supplements (ONS) vs. standard ONS (S-ONS) formulations remains insufficiently characterized. This study evaluated the cost impact of H-HEHP ONS vs. S-ONS in community-dwelling patients with frailty/sarcopenia and disease-related malnutrition (DRM) in France, using a cost calculator to estimate total annual costs and key drivers under current and alternative market-share scenarios.

**Methods:**

A cost analysis was conducted from the French public healthcare payer perspective over a 1-year time horizon. A deterministic Excel-based cost calculator, informed by a systematic literature review and national demographic and cost data, was used to estimate hospitalization-related costs associated with H-HEHP ONS vs. S-ONS in community-dwelling patients with frailty or sarcopenia and DRM. Current and alternative market-share scenarios were evaluated; robustness of results was assessed using one-way sensitivity analyses.

**Results:**

Treatment with H-HEHP ONS at the 2024 market share (73%) within the overall ONS market in community-dwelling patients with frailty/sarcopenia and DRM would generate annual cost savings of €17.4 million compared with S-ONS alone in the French community setting. Per-patient savings of €74 were driven primarily by shorter length of hospital stay and fewer readmissions. Increasing H-HEHP ONS market share to 80% in the community setting would generate an additional €1.7 million annually beyond current utilization.

**Conclusion:**

The cost savings identified in this analysis provide valuable information to guide healthcare decision making. As healthcare systems continue to balance competing priorities within constrained budgets, this study supports H-HEHP ONS as a cost-saving intervention, providing population data essential for informed formulary and reimbursement decisions.

## Introduction

Disease-related malnutrition (DRM) is a complex, multifactorial syndrome resulting from inadequate food intake, impaired nutrient absorption, or altered metabolism in acute or chronic disease (1). DRM represents an important public health challenge affecting millions of individuals worldwide. The syndrome is associated with reduced survival, increased complications, and the risk of hospitalization (1–4). These adverse outcomes are consistently observed across vulnerable populations, particularly among older adults with frailty and sarcopenia. DRM imposes a substantial economic burden across the European Union, estimated at €120–170 billion annually (5–7), highlighting the need for effective nutritional interventions in at-risk populations.

Studies across European healthcare settings demonstrate increased resource use and costs associated with DRM. In community-dwelling older adults in Spain, frailty was associated with significantly higher hospitalization rates (16.3% vs. 8.0% in non-frail older adults, *p* = 0.001) and 23.7% greater annual costs (8). Similarly, malnutrition or risk of malnutrition was independently associated with 11–13% higher hospitalization costs compared with well-nourished individuals (9). These effects persist beyond hospital discharge, with sarcopenia linked to a 2.25-fold higher risk of readmission compared with patients without sarcopenia (*p* = 0.03) (10).

Recognizing the substantial burden of DRM, major nutrition societies recommend oral nutritional supplements (ONS) for older adults with malnourishment, with French guidelines advising ≥400 kcal and ≥30 g of protein per day (11–13). A French study of community-dwelling older adults with malnourishment showed that ONS intake of ≥500 kcal/day or ≥30 g protein/day reduced hospitalization risk three- to five-fold, with healthcare costs significantly lower in compliant patients (€1,389 vs. €3,502, *p* = 0.042) (14). These findings align with broader European evidence demonstrating clinical and economic benefits of ONS (15–17).

Patients with DRM require increased energy and protein due to metabolic stress, yet experience reduced appetite, early satiety, and gastrointestinal symptoms that limit oral intake. Standard ONS (S-ONS) formulations require larger volumes to meet these needs, which are often poorly tolerated. Highest high-energy, high-protein (H-HEHP) ONS products, defined according to energy and protein thresholds consistent with French reimbursement criteria (18) (e.g., 1.8–2.4 kcal/mL and 10–14 g of protein per 100 mL), provide more concentrated nutrition than S-ONS formulations (≤1.5 kcal/mL; 3.25–10 g protein per 100 mL, with 10 g of protein representing the upper limit for S-ONS formulations, including some older high-energy, high-protein [HEHP] formulations), improving nutritional intake and patient compliance (19).

Economic evidence supports the cost-effectiveness of ONS in community settings. In France, a pharmaco-economic study demonstrated that ONS prescription was associated with cost savings of €723 per patient compared with infrequent prescription, driven primarily by reduced hospital care costs (€551) and nursing care savings (€145), alongside significant improvements in nutritional status as measured by the Mini Nutritional Assessment (MNA) scale (*p* < 0.005) (20). These findings are supported by subsequent community studies across Europe (21–24), demonstrating consistent cost savings and reduced hospitalizations.

Despite growing evidence supporting nutritional interventions, the comparative economic value of H-HEHP ONS vs. S-ONS remains insufficiently characterized, particularly in community settings where data on cost savings are limited but critical for informing formulary decisions, reimbursement policies, and procurement strategies.

This study compares the cost impact of H-HEHP ONS vs. S-ONS in community-dwelling patients with frailty/sarcopenia and DRM in France from a public healthcare system perspective. A customizable cost calculator informed by a systematic literature review was developed to estimate total annual costs and key drivers under varying market-share scenarios.

## Materials and methods

### Model structure, perspective, and time horizon

A Microsoft® Excel-based cost calculator was developed to estimate the cost impact of H-HEHP ONS compared with S-ONS in community-dwelling patients with frailty/sarcopenia and DRM, from the perspective of the French public healthcare system.

The cost calculator allows users to input local population parameters, treatment patterns, and cost inputs to generate estimates reflecting real-world practice patterns and reimbursement structures. Four scenarios, each assuming that only 40% of community-dwelling patients with frailty/sarcopenia and DRM receive ONS, were evaluated: no ONS usage, all eligible patients receiving S-ONS, a mix of S-ONS and H-HEHP ONS at a market share of 27% S-ONS/73% H-HEHP ONS (based on 2024 market data), and H-HEHP ONS at a projected increased relative market share vs. S-ONS of 80% (25). The projected market share was based on expected clinical uptake.

The analysis employed a 1-year time horizon and adopted a payer perspective, including direct costs associated with community ONS provision, healthcare resource utilization, and management of malnutrition-related complications. Discounting was not applied, consistent with standard methodology for short-term cost analyses. The model underwent internal validation, including formula verification, error checking, extreme value testing, and face validity assessment by health economists.

All calculations within the cost calculator were performed using source values to all reported decimal places. Values presented in this manuscript are rounded for reporting purposes; therefore, minor discrepancies may occur when results are independently recalculated using the rounded values presented in the tables. Specific rounding rules are provided for each results table.

### Analytical approach

Three analyses were conducted:

- A per-patient analysis, estimating costs and outcomes at the individual patient level
- A population-level (budget impact) analysis, extrapolating per-patient results to the target population
- A scenario analysis, reflecting increased uptake of H-HEHP ONS in the community setting

In addition, one-way deterministic sensitivity analyses were conducted to assess the robustness of results to variations in key inputs.

### Study population

The target population comprised community-dwelling patients aged ≥70 in France who were frail, had DRM, and were receiving ONS. Population size was derived using national demographic data from the DREES–INSEE LIVIA model, which projects dependent older adults by living situation (26). This population included individuals classified as GIR 3–4 and GIR 1–2 on the French autonomy scale (Groupe Iso-Ressources), corresponding to partial and full dependency, respectively, and reflecting a need for assistance with activities of daily living.

Based on Arnault et al. (27), frailty prevalence was 25.2% among individuals aged ≥75 years and 5.1% among those aged 70–74 years. These age-specific proportions were applied to the corresponding age-group population counts to estimate the number of frail individuals aged ≥70 years living at home. To derive the population with disease-related malnutrition, a malnutrition prevalence of 7.5% among community-dwelling individuals aged ≥70 years, as reported by institutional public health sources in France, was applied (28, 29).

In combination with national demographic data, information from Rives-Lange et al. (30) was used to derive the proportion of patients receiving nutritional support. This proportion was applied to the population aged ≥70 with frailty and DRM to estimate the numbers receiving and not receiving nutritional support. The proportion of patients receiving S-ONS and H-HEHP ONS was derived based on a market share of 27% S-ONS/73% H-HEHP ONS (based on 2024 market data) (Table 1).

**Table 1:** Study Population in France.

Parameters	Variable	Population size^§^	Source
Population over 70, living independently at home*	–	10,659,820	Direction de la Recherche, des Études, de l’Évaluation et des Statistiques (2025) (26)
Population over 70, living independently at home and suffers from frailty*	25.2% of population aged ≥75; 5.1% of population aged 70–75	1,899,609	Direction de la Recherche, des Études, de l’Évaluation et des Statistiques (2025) (26) Arnault et al. (2024) (27)
Population over 70, living independently at home, suffers from frailty and malnourishment	7.5%^†^ of individuals aged ≥70 at home are malnourished	799,487	Le Collectif de Lutte contre la Dénutrition (28) Ministère du Travail, de la Santé, des Solidarités et des Familles (2025) (29)
Of these, number of patients receiving nutritional support	40% of individuals receive ONS^‡^	319,795	Rives-Lange et al. (2025) (30)^†^
Number of patients receiving S-ONS	27% of individuals receive S-ONS	86,345	2024 market data
Number of patients receiving H-HEHP ONS	73% of individuals receive H-HEHP ONS	233,450	2024 market data

DRM, disease-related malnutrition; H-HEHP, highest high-energy, high-protein; ONS, oral nutritional supplements; S-ONS, standard oral nutritional supplements. *The population aged 70–74 was derived by proportionally dividing the 60–74 age group. †7.5% represents an estimate of the average proportion of individuals aged ≥70 living independently at home who are malnourished (28, 29). ‡Details of the calculations used to derive the 40% conversion factor are provided in the supplementary evidence (Study Population Calculations). § Calculations were performed in the cost calculator using source values to all reported decimal places; reported values were rounded to the nearest whole number for presentation.

### Interventions and treatment costs

Four ONS formulation products representing two formulation categories were included: two S-ONS products and two H-HEHP ONS products. S-ONS formulations were defined as providing ≤1.5 kcal/mL and 7–20 g protein per bottle, while H-HEHP ONS provided 1.8–2.5 kcal/mL and 20–28 g protein per bottle. These four products accounted for approximately 99% of 200 mL ONS use in France in 2024 (25). Community-setting treatment costs for these products are summarized in Table 2 and were based on official reimbursed prices in France (31).

**Table 2:** Cost of S-ONS and H-HEHP ONS formulations.

Product attributes							Frailty/sarcopenia and DRM		
Product type	Price per bottle (€)* (37)	Bottle size (mL)	kcal per bottle^†^	Protein per bottle (g)	Bottles required per day^‡^	Average cost per day (€)	Average length of use (days)	Average cost of nutrition per patient (€)	Current average usage of product^§^ (25)
H-HEHP ONS (10 g; HP > 10 g HE)	2.24	200	360–400	20	1.3	2.95	30	88.42	47%
H-HEHP ONS (14 g; HP ≥ 14 g HE conc.)	2.83	200	450–480	>28	1.1	3.04	30	91.29	26%
S-ONS (7 g; HP ≥ 7 g HE)	1.82	200	300	14–20	1.7	3.03	30	91.00	12%
S-ONS (juice style; carbohydrate and protein only)	1.98	200	250–300	7.5	1.8	3.60	30	108.00	14%

DRM, disease-related malnutrition; HE, high-energy; HEHP, high-energy, high-protein; H-HEHP, highest high-energy, high-protein; HP, high-protein; ONS, oral nutritional supplements; S-ONS, standard oral nutritional supplements; VAT, value added tax. *The price per bottle was derived from the 2024 reimbursement price, excluding VAT (33). †Midpoint used in the model. ‡Based on the goal of 500 kcal and 30 g protein per day supported by Séguy et al. (2020) (14). § The four products selected represent approximately 99% of 200 mL ONS products used in 2024 (25). In the cost calculator, the remaining 1% was assumed to receive an S-ONS product with a cost of €0. Products were categorized as S-ONS or H-HEHP ONS based on protein and energy thresholds aligned with French reimbursement criteria (18). H-HEHP ONS were defined as products providing 1.8–2.4 kcal/mL and 10–14 g protein per 100 mL, whereas S-ONS provided ≤1.5 kcal/mL and 3.25–10 g of protein per 100 mL. One product classified as S-ONS met earlier HEHP thresholds and was not considered H-HEHP under current definitions. Calculations were performed in the cost calculator using source values to all reported decimal places. For presentation purposes, reported values for bottles required per day were rounded to one decimal place, while average cost per day and average cost of nutrition per patient were rounded to two decimal places.

Daily bottle requirements were calculated to achieve a target of 500 kcal/day and ≥30 g protein/day. Although French guidelines specify a minimum of 400 kcal/day, the higher target was selected based on clinical evidence from Séguy et al. (14), which demonstrated that ONS intake ≥500 kcal/day significantly reduced hospitalization risk in community-dwelling malnourished older adults (*p* = 0.002). Although the average length of ONS use has been reported as 97 days (32), ONS duration in the cost calculator was set at 30 days, reflecting the minimum recommended treatment period in French clinical practice guidelines. The duration of ONS use was assumed to be equal across S-ONS and H-HEHP ONS scenarios in the cost calculator.

Treatment costs per patient were calculated by estimating daily nutrition costs (price × consumption) and applying duration of use, with weighted averages used where multiple products were included.

Market share assumptions reflected observed H-HEHP ONS share in France in 2024 (Table 2), based on manufacturer sales data and market research. An alternative scenario modeled increased H-HEHP ONS adoption, assuming 80% H-HEHP ONS and 20% S-ONS use in the community setting.

### Clinical outcomes and costs

Outcome data and associated costs comparing H-HEHP ONS with S-ONS were derived from published literature identified through a systematic literature review (SLR). Details of the SLR, including the selection criteria (Supplementary Table S1), search terms for the various databases (Supplementary Tables S2–S4), and the PRISMA flow diagram (Supplementary Figure S1), are provided in the supplementary evidence. For community-dwelling patients, the primary clinical outcome modeled was hospitalization, defined by hospital admission rate and length of stay, as identified from and selected based on SLR findings.

Hospitalization is a major consequence of DRM and a key cost driver in this analysis. For community-dwelling patients with frailty/sarcopenia and DRM, hospitalization rates and length of stay were sourced from published European epidemiological studies comparing malnourished and well-nourished older adults (17, 22). Evidence gaps were addressed by indirect treatment comparison methods. Further details are provided in the supplementary evidence.

Complication costs were applied based on the probability of hospitalization when receiving no ONS, S-ONS, or H-HEHP ONS and included hospital-related costs for managing post-operative events, infections, pressure ulcers, and diarrhea. The total per-patient costs for each ONS scenario were calculated as the sum of treatment costs and complication management costs (Table 3). Further methodological details regarding the per-patient cost calculations used to derive the results presented in Table 3 are provided in the supplementary evidence. The daily cost of hospitalization (cost of hospital bed per day) is provided in Supplementary Table S6.

**Table 3:** Treatment and complication costs per patient by ONS treatment.

Cost parameter	Without ONS (€)	S-ONS (€)	H-HEHP ONS (€)	Cost savings with H-HEHP ONS vs. S-ONS (€)
Patients with frailty/sarcopenia and DRM				
Treatment costs	0	96	89	7
Complication costs	2,376	366	299	67
Total	2,376	462	388	74

DRM, disease-related malnutrition; H-HEHP, highest high-energy, high-protein; ONS, oral nutritional supplements; S-ONS, standard oral nutritional supplements. All scenarios of ONS usage assume that 40% of the eligible patient population receive ONS (H-HEHP or S-ONS). Calculations were performed in the cost calculator using source values to all reported decimal places; reported values were rounded to the nearest whole number for presentation.

Population-level costs were estimated by applying per-patient costs to a cohort of 799,487 community dwellers with frailty/sarcopenia and DRM, with treatment and complication costs aggregated across no ONS, S-ONS, and H-HEHP ONS scenarios, and cost savings calculated as the difference in total costs between H-HEHP ONS and S-ONS (Table 4). Further methodological details regarding the population-level cost calculations used to derive the results presented in Table 4 are provided in the supplementary evidence.

**Table 4:** Treatment and Complication Cost Analysis (Population-Level).

Population	Cost parameter	Without ONS (€)	S-ONS only (€)	With H-HEHP ONS (at 2024 market share within ONS market*) (€)	Cost savings with H-HEHP ONS (at 2024 market share*) vs. SONS only (€)
Community dwellers with frailty/sarcopenia and DRM	Treatment costs	0	30,842,451	29,207,958	17,356,283
	Complication costs	1,899,796,973	1,256,937,493	1,241,215,704	
	Total	1,899,796,973	1,287,779,945	1,270,423,661	

DRM, disease-related malnutrition; H-HEHP, highest high-energy, high-protein; ONS, oral nutritional supplements; S-ONS, standard oral nutritional supplements. *Market share within ONS market: 73% H-HEHP ONS in community (2024 data) vs. 27% S-ONS. All scenarios of ONS usage assume that 40% of eligible patient population receive ONS (H-HEHP or S-ONS). Calculations were performed in the cost calculator using source values to all reported decimal places; reported values were rounded to the nearest whole number for presentation.

### Sensitivity analyses

One-way deterministic sensitivity analyses were conducted to examine uncertainty around key input parameters and the robustness of model results. In the absence of parameter-specific uncertainty values, key model inputs, including population size, treatment duration, clinical outcomes, and costs, were varied by ±20% from their base-case values. This range was selected as a pragmatic approach to assess the robustness of model outputs and should be interpreted as exploratory rather than as an empirically-derived confidence bound. The impact of parameter uncertainty on total costs and cost differences between scenarios was assessed to identify key drivers of model outcomes.

## Results

### Per-patient analysis

Treatment, complication, and total costs per patient by ONS treatment scenario are presented in Table 3. For patients with frailty/sarcopenia and DRM in the community setting, the use of H-HEHP ONS instead of S-ONS would generate annual cost savings of €74 per patient. These cost savings were driven primarily by reduced hospitalization rates.

### Population-level analysis

The number of community-dwelling patients aged >70 years with frailty/sarcopenia and DRM across ONS scenarios (no ONS, S-ONS, and H-HEHP ONS) is provided in Supplementary Table S7. The data reflect patient numbers under three treatment scenarios: no ONS availability (Scenario 1; 0% ONS uptake), availability of S-ONS only (Scenario 2; 40% ONS uptake), and availability of both S-ONS and H-HEHP ONS (Scenario 3; 40% ONS uptake, of whom 27% received S-ONS and 73% received H-HEHP ONS). To assess population-level budget impact, total costs were compared across treatment scenarios (Table 2). Treatment, complication, and total costs are presented in Table 4.

In the absence of ONS usage, annual costs associated with DRM-related complications in community-dwelling patients with frailty/sarcopenia in France totaled €1,899,796,973. When assuming use of S-ONS only, total costs encompassing both treatment and complication management were €1,287,779,945. Assuming H-HEHP ONS at the 2024 market share (Table 2), total costs were €1,270,423,661. Detailed cost breakdowns for each treatment scenario are provided in Table 4.

Comparing H-HEHP ONS at the 2024 relative market share (73%) with S-ONS alone revealed substantial cost savings. At the 2024 market share, the introduction of H-HEHP ONS would generate total annual cost savings of €17,356,283 among patients with frailty/sarcopenia and DRM in the community setting in France. The primary driver of cost savings with H-HEHP ONS was reduced hospitalization costs.

### Projected market share scenario analysis

To evaluate the potential impact of broader H-HEHP ONS adoption in the community setting, additional cost savings associated with increasing H-HEHP ONS relative market share vs. S-ONS from 73 to 80% within the overall ONS market were assessed. Results for this scenario are presented in Table 5. The analysis captures incremental cost savings from switching about a quarter (25.9%) of patients already receiving S-ONS to H-HEHP ONS; costs associated with patients not receiving ONS are therefore irrelevant.

**Table 5:** Projected cost analysis in patients currently receiving ONS.

Population	Cost parameter	With H-HEHP ONS at 2024 market share (€)	With H-HEHP ONS at projected 80% market share (€)	Additional savings (€)
Patients with frailty/sarcopenia and DRM	Treatment Costs	29,207,958	29,051,226	1,664,301
	Complication Costs	101,337,995	99,830,426	
	Total Costs	130,545,953	128,881,652	

DRM, disease-related malnutrition; H-HEHP, highest high-energy, high-protein; ONS, oral nutritional supplements; S-ONS, standard oral nutritional supplements. ONS overall market share: 40% of eligible patient population. Market share within overall ONS market: 73% H-HEHP ONS (2024 data) vs. 27% S-ONS. Projected scenario within overall ONS market: 80% H-HEHP ONS vs. 20% S-ONS. Calculations were performed in the cost calculator using source values to all reported decimal places; reported values were rounded to the nearest whole number for presentation.

Increasing H-HEHP ONS market share within the overall ONS market (40% of eligible patient population in the community setting) from 73 to 80% would result in additional annual savings of €1,664,301 (Table 5). As shown in Table 5, these incremental savings were driven by continued reductions in complication-related costs as a greater proportion of patients transition from S-ONS to H-HEHP ONS. The magnitude of savings reflects both the large number of community-dwelling patients who are suffering from frailty/sarcopenia and DRM and the per-patient cost differences between H-HEHP and standard formulations. Overall, increasing H-HEHP ONS market share to 80% within the ONS market in the community setting would generate meaningful additional cost savings for the French healthcare system, highlighting the economic value of strategies that promote H-HEHP ONS use among eligible patients with DRM.

### Sensitivity analysis

The sensitivity analysis demonstrated that cost savings associated with H-HEHP ONS were robust across all plausible parameter ranges tested. Hospital admission rates and length of stay had the greatest influence, followed by treatment-related parameters (energy content per bottle, hospital bed day cost, price per bottle). Parameters with the greatest impact on cost savings are presented in the tornado diagram provided in the supplementary evidence (Supplementary Figure S3). Overall, the consistency of results across sensitivity analyses supports the validity of the base case findings and confirms that H-HEHP ONS remains cost saving compared with S-ONS under a range of assumptions.

## Discussion

This cost analysis indicates that, in France, treatment with H-HEHP ONS in community-dwelling patients with frailty/sarcopenia and DRM at the 2024 market share is estimated to lead to annual cost savings of €17.4 million compared with S-ONS alone. Per-patient savings of €74 were observed, driven primarily by shorter hospital length of stay and fewer readmissions. These cost savings reflect reductions in complication-related hospitalizations and the clinical benefits of these H-HEHP formulations, which provide concentrated nutrition and require less volume to meet nutritional requirements. Cost savings may be underestimated as the average duration of ONS use in real-world practice is 97 days (32), whereas a conservative assumption of 30 days was applied in the cost calculator. The current analysis reflects ONS use in approximately 40% of the eligible population. Expanding access to the remaining patients with DRM who may benefit from nutritional support could result in additional cost savings, alongside potential improvements in patient outcomes. Given ongoing population ageing, the size of the eligible population with frailty/sarcopenia and DRM is unlikely to decline, suggesting that the clinical and economic impact of nutritional interventions may become increasingly important over time.

Our findings align with previous economic evaluations of S-ONS in community settings. A French prospective study demonstrated that ONS compliance in malnourished older adults decreased hospitalization risk without additional healthcare cost (14). A systematic review of community-based ONS use similarly confirmed reductions in hospitalizations and mortality (17). Earlier pharmaco-economic analyses in community-dwelling older populations reported cost savings associated with ONS use (20, 24). Evidence from hospital-based studies further support ONS benefits, including a US quality-improvement program that reduced 30-day readmissions by more than 50% and generated savings of USD $3,800 per patient (33, 34), as well as budget impact analyses from Latin America projecting substantial annual cost savings (35, 36). This analysis provides the first direct economic comparison between H-HEHP and S-ONS formulations in the community setting, quantifying differential cost savings attributable specifically to H-HEHP products.

Increasing H-HEHP ONS market share to 80% in the community setting would generate an additional €1.7 million annually beyond current utilization, highlighting an opportunity for further cost optimization and demonstrating that strategies promoting broader H-HEHP ONS adoption would yield meaningful economic returns for the French healthcare system. Cost savings suggest that implementation of systematic nutritional screening and early intervention protocols in community-based care could yield substantial returns while improving patient outcomes. These results support the prioritization of H-HEHP ONS in formularies and their integration into standardized care pathways for patients with DRM. H-HEHP ONS investment represents a cost-saving strategy rather than additional expenditure, supporting its prioritization in nutritional support policies for at-risk populations.

This analysis has several notable strengths that enhance the validity and applicability of findings. The comprehensive scope encompasses a distinct patient population (frailty/sarcopenia with DRM) in the community setting, evaluating both 2024 market conditions and projected adoption scenarios. Clinical and cost inputs were informed by a systematic literature review, with priority given to France-specific data and conservative assumptions applied where evidence was heterogeneous. The Excel-based cost calculator offers a transparent and accessible decision-support tool, with an adaptable framework that enables localization of population, treatment, and cost inputs to support scenario testing and reproducibility.

Despite these strengths, several limitations should be acknowledged. The patient population was derived using published sources and proportional assumptions, which may not fully capture real-world demographic variation within the target population. Country-level data were not available for patients aged 70–74; therefore, broader age-group data (60–74 age group) were proportionally divided to derive the relevant age-group estimates. This approach assumes a uniform distribution of individuals across age bands and does not account for age-related demographic effects including differences in population size resulting from natural mortality. The 1-year time horizon may underestimate longer-term cumulative benefits of improved nutritional status, although it provides a conservative estimate aligned with healthcare system budget cycles. The cost calculator assumed equal duration of ONS use across S-ONS and H-HEHP ONS scenarios. As longer treatment durations are observed in real-world practice (32), this assumption is likely conservative, and longer use would be expected to further increase the incremental cost savings associated with H-HEHP ONS. The payer perspective excludes broader societal costs such as caregiver burden and productivity losses. Sensitivity analyses were limited to ±20% parameter variation, reflecting a pragmatic approach to assessing model robustness in the absence of parameter-specific uncertainty values. As these ranges were not derived from empirical data, they should be treated as exploratory rather than as data-driven confidence bounds. However, cost savings remained robust across the tested ranges. Reliance on France-specific data may limit direct generalizability to other healthcare systems; however, the transparent methodology and adaptable calculator support application in other settings. The analysis focused on community-dwelling patients and did not capture potential savings in hospital or nursing home settings, nor quality-of-life benefits, suggesting that reported cost savings likely represent conservative estimates of the overall economic value of H-HEHP ONS compared with standard formulations.

Future research priorities should focus on strengthening the evidence base for H-HEHP ONS in patients with DRM. Cost-utility analyses incorporating quality-adjusted life years and patient-reported outcomes would provide comprehensive value assessment from payer and patient perspectives. Pragmatic trials and real-world studies using administrative claims data are needed to validate model estimates in routine practice, while longer-term studies extending beyond 5 years would better capture the cumulative benefits of sustained nutritional support. Subgroup analyses by disease stage and malnutrition severity may inform precision nutrition approaches.

In addition, extending the analysis to patients with cancer would be valuable, given the high prevalence of malnutrition, increased metabolic demands, and frequent use of ONS in this population. Evaluating the clinical and economic impact of H-HEHP ONS in oncology settings could support decision-making across a broader range of indications. Extension of this analysis to hospital and nursing home settings would further provide a more comprehensive assessment of the total economic impact of H-HEHP ONS across the care continuum. Finally, validation of the cost calculator in other European healthcare systems would enhance generalizability and support broader adoption of H-HEHP ONS as a cost-effective intervention.

## Conclusion

This cost analysis indicates that, in France, treatment with H-HEHP ONS in community-dwelling patients with frailty/sarcopenia and DRM would generate annual cost savings of €17.4 million, based on currently estimated 40% ONS uptake by the eligible patient population split across H-HEHP ONS and S-ONS at the 2024 market shares (73%/27%). Increasing to 80% H-HEHP ONS market share within the overall ONS market share (40% of eligible patient population) is projected to generate an additional €1.7 million in annual savings. These savings were primarily driven by reductions in hospital length of stay and readmission rates, which more than offset higher product acquisition costs. Given the consistent per-patient cost differentials between H-HEHP and S-ONS, similar cost-saving patterns are expected in hospital and nursing home settings.

The cost savings identified in this analysis provide valuable information to guide healthcare decision making. The customizable cost calculator developed for this analysis enables payers and policymakers to assess economic impact within local contexts, supporting informed formulary and resource allocation decisions. As healthcare systems continue to balance competing priorities within constrained budgets, this study supports H-HEHP ONS as a cost-saving intervention, providing population data essential for informed formulary and reimbursement decisions.

## Data Availability

The original contributions presented in the study are included in the article/Supplementary material, further inquiries can be directed to the corresponding author.

## References

[1] World Health Organization. Regional Office for Europe (2023). Disease-related malnutrition: a time for action. Available online at: https://www.who.int/europe/publications/i/item/WHO-EURO-2023-8931-48703-72392 (Accessed 18 May 2026)

[2] Camilleri GM Delrieu L Bouleuc C Pierga JY Cottu P Berger F . Prevalence and survival implications of malnutrition and sarcopenia in metastatic breast cancer: a longitudinal analysis. Clin Nutr. (2024) 43:1710–8. doi: 10.1016/j.clnu.2024.06.014, 38908032

[3] Saroul N Puechmaille M Lambert C Hassan AS Biau J Lapeyre M . Prognosis in head and neck Cancer: importance of nutritional and biological inflammatory status. Otolaryngol Head Neck Surg. (2022) 166:118–27. doi: 10.1177/01945998211004592, 33845660

[4] Rossi AP Scalfi L Abete P Bo M Cherubini A Corica F . Controlling nutritional status score as a predictor of mortality and hospitalization risk in elderly hospitalized men and women: the interplay with sarcopenia and disability. The GLISTEN study group. Clin Nutr ESPEN. (2023) 58:471. doi: 10.1016/j.clnesp.2023.09.138

[5] Freijer K Tan SS Koopmanschap MA Meijers JMM Halfens RJG Nuijten MJC. The economic costs of disease related malnutrition. Clin Nutr. (2013) 32:136–41. doi: 10.1016/j.clnu.2012.06.009, 22789931

[6] Ljungqvist O Man F. Under nutrition: a major health problem in Europe. Nutr Hosp. (2009) 24:369–70. 19721916

[7] Ljungqvist O van Gossum A Sanz ML de Man F. The European fight against malnutrition. Clin Nutr. (2010) 29:149–50. doi: 10.1016/j.clnu.2009.10.004, 19897284

[8] Alvarez-Bustos A Rodriguez-Sanchez B Carnicero-Carreno JA Sepulveda-Loyola W Garcia-Garcia FJ Rodriguez-Manas L. Healthcare cost expenditures associated to frailty and sarcopenia. BMC Geriatr. (2022) 22:747. doi: 10.1186/s12877-022-03328-0 PMC9469617 36096728

[9] Rodriguez-Manas L Rodriguez-Sanchez B Carnicero JA Rueda R Garcia-Garcia FJ Pereira SL . Impact of nutritional status according to GLIM criteria on the risk of incident frailty and mortality in community-dwelling older adults. Clin Nutr. (2021) 40:1192–8. doi: 10.1016/j.clnu.2020.07.032, 32826110

[10] Ballesteros-Pomar MD Gajete-Martin LM Pintor-de-la-Maza B Gonzalez-Arnaiz E Gonzalez-Roza L Garcia-Perez MP . Disease-related malnutrition and sarcopenia predict worse outcome in medical inpatients: a cohort study. Nutrients. (2021) 13. doi: 10.3390/nu13093194 PMC8465264 34578815

[11] Haute Autorité de Santé (HAS) (2007). Stratégie de prise en charge en cas de dénutrition protéino-énergétique chez la personne âgée, Available online at: https://www.has-sante.fr/jcms/c_546549/fr/strategie-de-prise-en-charge-en-cas-de-denutrition-proteino-energetique-chez-la-personne-agee (Accessed 18 May 2026).

[12] Haute Autorité de Santé (HAS) (2021). Diagnostic de la dénutrition chez la personne de 70 ans et plus. Recommandations de bonne pratique. Available online at: https://www.has-sante.fr/jcms/p_3165944/fr/diagnostic-de-la-denutrition-chez-la-personne-de-70-ans-et-plus (Accessed 18 May 2026)

[13] Delarue J Desport JC Dubern B Joly F Mas E Pitard A . Diagnosing undernutrition children and adults: new French criteria. Why, for what and for whom? A joint statement of the French National Authority for health and French Federation of Nutrition. Br J Nutr. (2022) 127:739–51. doi: 10.1017/S0007114521003537 33958009

[14] Seguy D Hubert H Robert J Meunier JP Guerin O Raynaud-Simon A. Compliance to oral nutritional supplementation decreases the risk of hospitalisation in malnourished older adults without extra health care cost: prospective observational cohort study. Clin Nutr. (2020) 39:1900–7. doi: 10.1016/j.clnu.2019.08.005, 31471163

[15] Cereda E Cappello S Colombo S Klersy C Imarisio I Turri A . Nutritional counseling with or without systematic use of oral nutritional supplements in head and neck cancer patients undergoing radiotherapy. Radiother Oncol. (2018) 126:81–8. doi: 10.1016/j.radonc.2017.10.015, 29111172

[16] Elia M Normand C Norman K Laviano A. A systematic review of the cost and cost effectiveness of using standard oral nutritional supplements in the hospital setting. Clin Nutr. (2016) 35:370–80. doi: 10.1016/j.clnu.2015.02.016, 26123475

[17] Cawood AL Burden ST Smith T Stratton RJ. A systematic review and meta-analysis of the effects of community use of oral nutritional supplements on clinical outcomes. Ageing Res Rev. (2023) 88:101953. doi: 10.1016/j.arr.2023.101953 37182743

[18] Legifrance. Arrêté du 7 mai 2019 portant modification de la procédure d’inscription et des conditions de prise en charge des produits pour complémentation nutritionnelle orale destinés aux adultes inscrits sur la liste des produits et prestations remboursables prévue à l’article L. 165-1 du code de la sécurité sociale. NOR: SSAS1913497A. Journal officiel, 10 Mai (2019) Available online at: https://www.legifrance.gouv.fr/download/pdf?id=-Ka05xQ22HeMQrELEmpe3aFvGs9WK7QfWI-0Yygfd_k=. (Accessed 02 June 2026).

[19] Nutricia. Disease related malnutrition and frailty. (2026). Available online at: https://www.nutricia.com/products/frailty-and-drm.html. (Accessed 18 May 2026).

[20] Arnaud-Battandier F Malvy D Jeandel C Schmitt C Aussage P Beaufrere B . Use of oral supplements in malnourished elderly patients living in the community: a pharmaco-economic study. Clin Nutr. (2004) 23:1096–103. doi: 10.1016/j.clnu.2004.02.003 15380901

[21] Nuijten M Mittendorf T. Gesundheitsökonomische Analyse der medizinischen Trinknahrungen in Deutschland. Aktuelle Ernährungsmedizin. (2012) 37:126–33. doi: 10.1055/s-0032-1306303, 22565334

[22] Brown F Fry G Cawood A Stratton R. Economic impact of implementing malnutrition screening and nutritional Management in Older Adults in general practice. J Nutr Health Aging. (2020) 24:305–11. doi: 10.1007/s12603-020-1313-3, 32115612 PMC7064449

[23] Smith TR Cawood AL Walters ER Guildford N Stratton RJ. Ready-made oral nutritional supplements improve nutritional outcomes and reduce health care use-a randomised trial in older malnourished people in primary care. Nutrients. (2020) 12:517. doi: 10.3390/nu12020512 PMC7071441 32085537

[24] Freijer K Nuijten MJ Schols JM. The budget impact of oral nutritional supplements for disease related malnutrition in elderly in the community setting. Front Pharmacol. (2012) 3:78. doi: 10.3389/fphar.2012.00078, 22629244 PMC3355295

[25] Caisse Nationale de l'Assurance Maladie. Dispositifs médicaux inscrits à la liste des produits et prestations - LPP'AM - 2016 à 2025 (2026). Available online at: https://www.assurance-maladie.ameli.fr/etudes-et-donnees/lpp-lppam. (Accessed 02 Jan 2026).

[26] Direction de la Recherche, des Études, de l’Évaluation et des Statistiques (DREES). Projections du nombre de personnes âgées en perte d'autonomie par lieu de vie (modèle LIVIA) (2025). Available online at: https://data.drees.solidarites-sante.gouv.fr/explore/dataset/projections-du-nombre-de-personnes-agees-en-perte-dautonomie-par-lieu-de-vie-mod/information/ (Accessed 23 March 2026).

[27] Arnault L Jusot F Renaud T La fragilité chez les 50 ans et plus en France et en Europe. Deux décennies d’évolution et d’inégalités à partir des données de l’enquête SHARE Available online at: (2024) https://share.dauphine.fr/fileadmin/mediatheque/share/documents/DNSS_num4.pdf. (Accessed 23 March 2026)

[28] Le Collectif de Lutte contre la Dénutrition (CLD). La dénutrition en chiffres. Available online at: https://www.luttecontreladenutrition.fr/la-denutrition-en-chiffres/. (Accessed 23 March 2026).

[29] Ministère du Travail, de la Santé, des Solidarités et des Familles Parole d’experte: comprendre la dénutrition chez les personnes âgées (2025) https://solidarites.gouv.fr/parole-dexperte-comprendre-la-denutrition-chez-les-personnes-agees. (Accessed 23 March 2026)

[30] Rives-Lange C Desplas D Zureik M. Ambulatory artificial nutrition support use in adolescents and adults: a nationwide population-based descriptive cohort study. JPEN J Parenter Enteral Nutr. (2026) 50:67–76. doi: 10.1002/jpen.70022, 41153070

[31] Ministère de la Santé et de la Prévention. Avis relatif à la tarification des produits pour complémentation nutritionnelle orale destinés aux adultes visés à l'article L. 165-1 du code de la sécurité sociale (2019) Available online at: https://www.legifrance.gouv.fr/jorf/id/JORFTEXT000038457553. (Accessed 16 Dec 2025)

[32] Raynaud-Simon A. Delsart D. Revel E. Salhi A. Bugnard F. Avrillier-Rambourg J. Evolution des remboursements de CNO par l’assurance maladie entre 2018 et 2022 en France: une étude en vie réelle sur l’Echantillon du Système National des Données de santé. (ESND). Oral presentation presented at Société Francophone de Nutrition Clinique et Métabolisme (SFNCM), 10-12 December (2025)

[33] Sriram K Sulo S VanDerBosch G Partridge J Feldstein J Hegazi RA . A comprehensive nutrition-focused quality improvement program reduces 30-day readmissions and length of stay in hospitalized patients. JPEN J Parenter Enteral Nutr. (2017) 41:384–91. doi: 10.1177/0148607116681468, 27923890

[34] Sulo S Feldstein J Partridge J Schwander B Sriram K Summerfelt WT. Budget impact of a comprehensive nutrition-focused quality improvement program for malnourished hospitalized patients. Am Health Drug Benefits. (2017) 10:262–70. PMC5620506 28975010

[35] Buitrago G Vargas J Sulo S Partridge JS Guevara-Nieto M Gomez G . Targeting malnutrition: nutrition programs yield cost savings for hospitalized patients. Clin Nutr. (2020) 39:2896–901. doi: 10.1016/j.clnu.2020.01.020 31917050

[36] Sulo S Vargas J Gomez G Misas JD Serralde-Zuniga AE Correia M. Hospital nutrition care informs potential cost-savings for healthcare: a budget impact analysis. Clin Nutr ESPEN. (2021) 42:195–200. doi: 10.1016/j.clnesp.2021.01.019, 33745577

[37] Ministère de la Santé et de la Prévention. Arrêté du 7 mai 2019 portant modification de la procédure d'inscription et des conditions de prise en charge des produits pour complémentation nutritionnelle orale destinés aux adultes inscrits sur la liste des produits et prestations remboursables prévue à l'article L. 165-1 du code de la sécurité sociale (2019) Available online at: https://www.legifrance.gouv.fr/jorf/id/JORFTEXT000038456507 (Accessed 02 Jan 2026)

