# Supplementary material for: Economic value of high-energy, high-protein oral nutritional supplements in disease-related malnutrition in the French community setting
Source: Front Nutr. 2026 Oct 14;13:1952501. doi: 10.3389/fnut.2026.1952501 (PMC13713391; doi:10.3389/fnut.2026.1952501)
Supplement: Supplementary file 1 [file Supplementary_file_1.docx]

Economic Value of High-Energy, High-Protein Oral Nutritional Supplements in Disease-Related Malnutrition in the French Community Setting

# Supplementary Evidence

## Study Population Calculations

Information from Rives-Lange et al. (2025) was used to facilitate the derivation of the population of individuals aged ≥70, with frailty/sarcopenia, who are living at home with malnourishment.^1^ To determine the total population aged ≥70 in the ONS group described by Rives-Lange et al. (2025), the 60–74 age group was divided into three equal 5-year bands (60–64, 65–69, and 70–74), and combined with the 75+ age group. Based on this approach, 768,097 individuals aged ≥70 were estimated to be receiving ONS in the Rives-Lange et al. (2025) publication.^1^ The proportion of patients receiving ONS was derived by comparing the estimated number of individuals aged ≥70 receiving ONS (n=768,097) with the total frail, community‑dwelling population aged ≥70 in the current analysis (n=1,899,609), yielding 40%. This proportion was then applied to population of individuals aged ≥70 who are frail, living at home, and malnourished (n=799,487) to estimate the number receiving ONS (n=319,795).

## Systematic Literature Review

Table S1: Eligibility Criteria Used in the Search Strategy

| Category | Inclusion criteria | Exclusion criteria |
| --- | --- | --- |
| Population | Adults ≥18 years old with:   - DRM - Frailty/sarcopenia - Cancer who are experiencing disease-related malnutrition (DRM)   Setting:   - Hospital - Community - Nursing homes | - Patients with other diseases only - Frailty/sarcopenia without malnutrition - Cancer without malnutrition |
| Intervention/comparator | No restriction   - Studies of special interest are those reporting economic benefit of medical nutrition (MN) with a focus on high-energy, high-protein oral nutritional supplements (HEHP ONS) | - Dietary counselling only - Studies of non-ONS interventions, such as those with less than two macronutrients, no micronutrients |
| Outcomes | **Economic evaluations (models and trial based)**   - Model summary (including perspective, time horizon, discounting and model inputs) and structure, where applicable - Assumptions underpinning model structures. - Summary health outcomes (e.g. quality adjusted life years [QALYs}, disability-adjusted life years (DALYs), life years gained [LYG]) - Incremental cost effectiveness ratios (ICERs): cost per QALY/DALY/LYG, cost per event avoided - Sources of clinical, cost and quality of life inputs, mood, patient preferences etc   **Cost/resource use**   - Direct/indirect costs, estimates of healthcare resource use, length of stay, cost drivers including hospitalization and length of stay, assumptions underpinning resource use, methods of valuation   **Health state utility values**   - Utilities derived using generic preference-based instruments (e.g. EQ-5D, SF-6D, HUI2, HUI3, AQoL) for relevant health states - Direct utility estimates (e.g. standard gamble, time trade off)   **Clinical burden**   - Disease progression - Natural history - Clinical/treatment guidelines - Treatment patterns   **Humanistic burden**   - Health-related quality of life (HRQoL) or patient reported outcomes assessing quality of life, mobility, social wellbeing | - Clinical efficacy - Epidemiology - Pharmacokinetics/ pharmacovigilance outcomes - In vitro outcomes - Studies of methodological approach to deriving generic utility scores, that do not report utility values for the population of interest |
| Study design / setting | - Any study design that reports primary data - Systematic literature reviews (SLRs) or meta-analyses will be used for reference checking only | - Animal / in-vitro studies - Editorials - Letters - Case studies/reports - Narrative reviews |
| Language of publication | - English language - Non-English language publications from countries of interest will be tagged and considered for possible inclusion in consultation with Nutricia | - |
| Date of publication | - Full texts 2015 onwards - Conference abstracts 2022 onwards | - Full texts prior to 2015 - Conference abstracts prior to 2022 |
| Countries | - UK, NL, FR, SW, SP, IT and with a focus on FR) | - Other countries not listed |

Abbreviations: DALY, disability-adjusted life years; DRM, disease-related malnutrition; ICER, Incremental cost effectiveness ratios; LYG, life year gained; MN, medical nutrition; ONS, oral nutritional supplement; SLR, systematic literature review; SW, Sweden; QALY, quality adjusted life year

Table S2: Embase Search (1980 to 2024 Week 46)

| **#** | **Search term** | **Results** |
| --- | --- | --- |
| 1 | sarcopenia.mp. or exp sarcopenia/ | 33,481 |
| 2 | muscle atrophy.mp. or *muscle atrophy/ | 46,663 |
| 3 | exp frailty/ or frailty.mp. | 52,071 |
| 4 | exp oncology/ or exp malignant neoplasm/ or (Cancer or neoplasm or oncology or tumo?r or metasta$).mp. | 6,919,362 |
| 5 | 1 or 2 or 3 or 4 | 7,018,303 |
| 6 | *nutrition/ or malnutrition/ or *nutritional status/ or *nutritional assessment/ | 160,118 |
| 7 | (malnutrition or malnourish* or undernutrition or nutritional deficienc* or nutrition).ti. | 107,295 |
| 8 | (((diet* or nutri*) adj3 supplement*) or ((Liquid or drink or sip or sip feed or feed or formula) adj3 (supplement* or nutrition*)) or (protein$ adj1 supplement$)).tw. | 102,410 |
| 9 | ((high energy or high protein) adj5 supplement).mp. | 172 |
| 10 | (high energy high protein or hehp).mp. | 83 |
| 11 | 5 and (6 or 7 or 8 or 9 or 10) [Population of interest] | 46,178 |
| 12 | exp "cost of illness"/ or (cost analysis or cost-analysis).ti,ab,kw. or ((disease$ or sickness or illness) adj2 cost$).ti,ab,kw. or (economic adj5 (burden or cost$ or impact)).ti,ab,kw. or ((direct or medical or indirect or nonmedical or societ* or employe*) adj2 (cost* or resource* or benefit*)).ti,ab,kw. or ((drug$ or healthcare or health care) adj2 (cost$ or price$ or pricing)).ti,ab,kw. or ((hospital$ or re-hospital$ or rehospital$ or admit$ or admission$ or re-admission$ or readmission$) adj2 (cost$ or price$ or rate$ or risk$ or number$)).ti,ab,kw. or length of stay.ab. or exp health care utilization/ or exp drug utilization/ or ((health care or healthcare or resource*) adj2 (use* or utili*)).ti,ab,kw. or exp *productivity/ or (productivity cost$ or productivity).ti,ab,kw. or (productivity loss or (productivity adj2 los*)).ti,ab,kw. or exp *Absenteeism/ or exp *Presenteeism/ or (absenteeism or presenteeism or sick leave).ti,ab,kw. | 1,270,008 |
| 13 | (EuroQOL 5-Dimension or Euroqol 5D or EQ-5D or EQ5D or Euroqol).mp. [mp=title, abstract, heading word, drug trade name, original title, device manufacturer, drug manufacturer, device trade name, keyword heading word, floating subheading word, candidate term word] | 34,111 |
| 14 | (Health utilities index or HUI).mp. [mp=title, abstract, heading word, drug trade name, original title, device manufacturer, drug manufacturer, device trade name, keyword heading word, floating subheading word, candidate term word] | 4,507 |
| 15 | (time trade off or time trade-off or ("TTO" adj2 "time trade")).mp. [mp=title, abstract, heading word, drug trade name, original title, device manufacturer, drug manufacturer, device trade name, keyword heading word, floating subheading word, candidate term word] | 2,330 |
| 16 | (short form 6D or short-form 6D).mp. [mp=title, abstract, heading word, drug trade name, original title, device manufacturer, drug manufacturer, device trade name, keyword heading word, floating subheading word, candidate term word] | 375 |
| 17 | (standard gamble or ("SG" adj2 "standard gamble")).mp. [mp=title, abstract, heading word, drug trade name, original title, device manufacturer, drug manufacturer, device trade name, keyword heading word, floating subheading word, candidate term word] | 1,261 |
| 18 | (15D or 16D or 17D).mp. [mp=title, abstract, heading word, drug trade name, original title, device manufacturer, drug manufacturer, device trade name, keyword heading word, floating subheading word, candidate term word] | 4,966 |
| 19 | exp short form 12/ or exp short form 20/ or exp short form 36/ | 54,627 |
| 20 | ("quality of well-being index" or "quality of wellbeing index" or "quality of well being index").mp. [mp=title, abstract, heading word, drug trade name, original title, device manufacturer, drug manufacturer, device trade name, keyword heading word, floating subheading word, candidate term word] | 25 |
| 21 | (medical outcome adj1 (survey or stud*)).mp. [mp=title, abstract, heading word, drug trade name, original title, device manufacturer, drug manufacturer, device trade name, keyword heading word, floating subheading word, candidate term word] | 1,313 |
| 22 | or/13-21 | 97,066 |
| 23 | (QoL or HRQoL or HRQL).mp. [mp=title, abstract, heading word, drug trade name, original title, device manufacturer, drug manufacturer, device trade name, keyword heading word, floating subheading word, candidate term word] | 151,719 |
| 24 | exp "quality of life"/ | 726,068 |
| 25 | (health related quality of life or health-related quality of life).mp. | 95,680 |
| 26 | ((quality of life or QoL) adj10 (question$ or instrument or scale$1or score$1)).mp. [mp=title, abstract, heading word, drug trade name, original title, device manufacturer, drug manufacturer, device trade name, keyword heading word, floating subheading word, candidate term word] | 184,073 |
| 27 | or/23-26 | 759,734 |
| 28 | health state$.mp. | 15,677 |
| 29 | utilit*.mp. | 415,226 |
| 30 | Patient Preference/ or preference.mp. | 206,958 |
| 31 | (map$ or regression).mp. [mp=title, abstract, heading word, drug trade name, original title, device manufacturer, drug manufacturer, device trade name, keyword heading word, floating subheading word, candidate term word] | 2,619,996 |
| 32 | exp health status/ | 344,221 |
| 33 | health survey/ | 240,824 |
| 34 | exp daily life activity/ | 124,041 |
| 35 | ("Activities of Daily Living" or "IADL").mp. [mp=title, abstract, heading word, drug trade name, original title, device manufacturer, drug manufacturer, device trade name, keyword heading word, floating subheading word, candidate term word] | 57,159 |
| 36 | Psychometrics.mp. or exp psychometry/ | 120,532 |
| 37 | ("health year equivalent" or "HYE").mp. [mp=title, abstract, heading word, drug trade name, original title, device manufacturer, drug manufacturer, device trade name, keyword heading word, floating subheading word, candidate term word] | 176 |
| 38 | or/28-37 | 3,844,480 |
| 39 | 27 and 38 | 187,705 |
| 40 | 22 or 39 [QoL filter] | 252,957 |
| 41 | *observational study/ or *Case control study/ or *Longitudinal study/ or *Retrospective study/ or *cross-sectional study/ or *Cohort analysis/ or ((cohort or case control or follow up or observational or longitudinal or retrospective or cross sectional) adj (study or studies)).ti,ab. or (Cohort analy$ or Case control or longitudinal or retrospective or cross sectional).tw. or register/ or registr*.ti,ab. or ((real adj3 world) or ((real-world or real world) adj2 (data or evidence or research or study or studies or trial*))).ti,ab. or (((medical or database*) adj3 claim*) or (hospital adj3 record*)).ti,ab. or prognosis.ti,ab. or natural history.ti,ab. [Clinical burden filter] | 4,534,401 |
| 42 | exp economic model/ or exp economic evaluation/ or exp "cost effectiveness analysis"/ or ((cost-effective* adj1 analys*) or (cost adj1 effectiveness adj1 analys*)).mp. or exp "cost benefit analysis"/ or ((cost benefit adj1 analys*) or (cost-benefit adj1 analys*)).mp. or exp "cost utility analysis"/ or (cost utility analys* or (cost-utility adj1 analys*)).mp. or exp "cost minimization analysis"/ or (cost minimi?ation analys* or (cost-minimi?ation adj1 analys*)).mp. or (cost consequence analys* or (cost consequence adj1 analys*)).mp. | 380,411 |
| 43 | ((economic or pharmacoeconomic) adj2 (evaluation or assessment or analysis or stud*)).mp. and (("CEA" or "CMA" or "CBA" or "CUA" or "CCA" or "CEM").mp. or exp decision theory/ or exp decision tree/ or decision tree.mp. or economic model.mp. or (markov or deterministic or monte carlo or partition* survival).mp. or ((transition adj1 probabilit*) or (health adj1 stat*) or (sensitivity adj1 analys*) or (health adj1 outcome)).mp. or ((patient level or patient-level or discrete event or discrete-event) adj1 simulat*).mp. or (incremental-cost or incremental cost).mp. or ("ICER" or "QALY" or "DALY" or "WTP" or "TTO").mp.) | 17,444 |
| 44 | 42 or 43 [Economic evaluation filter] | 382,087 |
| 45 | 12 or 40 or 41 or 44 [burden outcomes / study design filters] | 5,770,975 |
| 46 | 11 and 45 | 13,853 |
| 47 | exp Netherlands/ or exp Italy/ or exp Sweden/ or exp France/ or exp Spain/ or exp United Kingdom/ or (EU5 or europe or united kingdom or England or english or Scotland or scottish wales or welsh or Ireland or Irish or Spain or Spanish or france or french or italy or italian or sweden or swedish or netherlands or dutch).mp,ad,cp,gc. | 18,141,764 |
| 48 | 46 and 47 | 7,810 |
| 49 | exp animal/ not (exp human/ and exp animal/) | 4,938,785 |
| 50 | exp note/ or exp editorial/ or exp letter/ or exp case report/ or exp Case study/ or exp Abstract report/ | 5,731,582 |
| 51 | 48 not (49 or 50) | 7,332 |
| 52 | limit 51 to conference abstracts | 3,235 |
| 53 | limit 52 to yr="2020 -Current" | 1,614 |
| 54 | 51 not 52 [full text only] | 4,097 |
| 55 | limit 54 to yr="2015 -Current" | 2,886 |
| 56 | 53 or 55 [Full texts last 10 years and abstracts last 3 years] | 4,500 |
| 57 | limit 52 to yr="2015 -Current" | 2,867 |

Table S3: Ovid MEDLINE Search (1946 to November 15, 2024)

| **#** | **Search term** | **Results** |
| --- | --- | --- |
| 1 | sarcopenia.mp. or exp Sarcopenia/ | 20,717 |
| 2 | muscle atrophy.mp. or *Muscular Atrophy/ | 16,697 |
| 3 | frailty.mp. or exp Frailty/ | 30,547 |
| 4 | (Cancer or neoplasm or oncology or tumo?r or metasta$).mp. or exp Neoplasms/ | 5,349,999 |
| 5 | 1 or 2 or 3 or 4 | 5,404,525 |
| 6 | *Nutrition Therapy/ or exp malnutrition/ or *Nutritional Status/ or *Nutrition Assessment/ | 169,906 |
| 7 | (malnutrition or malnourish* or undernutrition or nutritional deficienc* or nutrition).ti. | 95,731 |
| 8 | (((diet* or nutri*) adj3 supplement*) or ((Liquid or drink or sip or sip feed or feed or formula) adj3 (supplement* or nutrition*)) or (protein$ adj2 supplement$)).tw. | 84,509 |
| 9 | ((high energy or high protein) adj5 supplement).mp. | 134 |
| 10 | (high energy high protein or hehp).mp. | 68 |
| 11 | 5 and (6 or 7 or 8 or 9 or 10) [Population of interest] | 30,466 |
| 12 | exp "cost of illness"/ or exp "Costs and Cost Analysis"/ or (cost analysis or cost-analysis).ti,ab,kw. or ((disease$ or sickness or illness) adj2 cost$).ti,ab,kw. or (economic adj5 (burden or cost$ or impact)).ti,ab,kw. or ((health care or healthcare or resource*) adj2 (use* or utili*)).ti,ab,kw. or ((direct or medical or indirect or nonmedical or societ* or employe*) adj2 (cost* or resource* or benefit*)).ti,ab,kw. or (cost$ or price$ or pricing).ti,ab,kw. or exp Drug Costs/ or exp Hospital Costs/ or Length of Stay/ or length of stay.ti,ab,kw. or exp Health Care Costs/ or ((fiscal or funding or financ$) adj2 (data or health care)).mp. or (Fees.mp. and Charges/) or Budgets/ or ((caregiver* or carer*) adj2 burden).ti,ab,kw. or exp *productivity/ or (productivity cost$ or productivity).ti,ab,kw. or (productivity loss or (productivity adj2 los*)).ti,ab,kw. or *Absenteeism/ or *Presenteeism/ or *Medical Leave/ or (absenteeism or presenteeism or sick leave).ti,ab,kw. or medication cost.mp. [Economic burden] | 1,321,357 |
| 13 | (EuroQOL 5-Dimension or Euroqol 5D or EQ-5D or EQ5D or Euroqol).mp. [mp=title, book title, abstract, original title, name of substance word, subject heading word, floating sub-heading word, keyword heading word, organism supplementary concept word, protocol supplementary concept word, rare disease supplementary concept word, unique identifier, synonyms, population supplementary concept word, anatomy supplementary concept word] | 18,608 |
| 14 | (Health utilities index or HUI).mp. [mp=title, book title, abstract, original title, name of substance word, subject heading word, floating sub-heading word, keyword heading word, organism supplementary concept word, protocol supplementary concept word, rare disease supplementary concept word, unique identifier, synonyms, population supplementary concept word, anatomy supplementary concept word] | 2,300 |
| 15 | (time trade off or time trade-off or ("TTO" adj2 "time trade")).mp. [mp=title, book title, abstract, original title, name of substance word, subject heading word, floating sub-heading word, keyword heading word, organism supplementary concept word, protocol supplementary concept word, rare disease supplementary concept word, unique identifier, synonyms, population supplementary concept word, anatomy supplementary concept word] | 1,468 |
| 16 | (short form 6D or short-form 6D).mp. [mp=title, book title, abstract, original title, name of substance word, subject heading word, floating sub-heading word, keyword heading word, organism supplementary concept word, protocol supplementary concept word, rare disease supplementary concept word, unique identifier, synonyms, population supplementary concept word, anatomy supplementary concept word] | 164 |
| 17 | (standard gamble or ("SG" adj2 "standard gamble")).mp. [mp=title, book title, abstract, original title, name of substance word, subject heading word, floating sub-heading word, keyword heading word, organism supplementary concept word, protocol supplementary concept word, rare disease supplementary concept word, unique identifier, synonyms, population supplementary concept word, anatomy supplementary concept word] | 921 |
| 18 | (15D or 16D or 17D).mp. [mp=title, book title, abstract, original title, name of substance word, subject heading word, floating sub-heading word, keyword heading word, organism supplementary concept word, protocol supplementary concept word, rare disease supplementary concept word, unique identifier, synonyms, population supplementary concept word, anatomy supplementary concept word] | 3,403 |
| 19 | (short form 36 or shortform 36 or SF-36 or SF36 or SF 36).mp. [mp=title, book title, abstract, original title, name of substance word, subject heading word, floating sub-heading word, keyword heading word, organism supplementary concept word, protocol supplementary concept word, rare disease supplementary concept word, unique identifier, synonyms, population supplementary concept word, anatomy supplementary concept word] | 32,384 |
| 20 | (short form 12 or shortform 12 or SF12 or SF-12 or SF 12).mp. [mp=title, book title, abstract, original title, name of substance word, subject heading word, floating sub-heading word, keyword heading word, organism supplementary concept word, protocol supplementary concept word, rare disease supplementary concept word, unique identifier, synonyms, population supplementary concept word, anatomy supplementary concept word] | 8,297 |
| 21 | (medical outcomes survey or MOS).mp. [mp=title, book title, abstract, original title, name of substance word, subject heading word, floating sub-heading word, keyword heading word, organism supplementary concept word, protocol supplementary concept word, rare disease supplementary concept word, unique identifier, synonyms, population supplementary concept word, anatomy supplementary concept word] | 11,464 |
| 22 | (Quality of wellbeing index or QWB).mp. [mp=title, book title, abstract, original title, name of substance word, subject heading word, floating sub-heading word, keyword heading word, organism supplementary concept word, protocol supplementary concept word, rare disease supplementary concept word, unique identifier, synonyms, population supplementary concept word, anatomy supplementary concept word] | 219 |
| 23 | or/13-22 | 73,675 |
| 24 | (QoL or HRQoL or HRQL).mp. [mp=title, book title, abstract, original title, name of substance word, subject heading word, floating sub-heading word, keyword heading word, organism supplementary concept word, protocol supplementary concept word, rare disease supplementary concept word, unique identifier, synonyms, population supplementary concept word, anatomy supplementary concept word] | 85,055 |
| 25 | quality of life.mp. or exp "Quality of Life"/ | 495,150 |
| 26 | (health related quality of life or health-related quality of life).mp. [mp=title, book title, abstract, original title, name of substance word, subject heading word, floating sub-heading word, keyword heading word, organism supplementary concept word, protocol supplementary concept word, rare disease supplementary concept word, unique identifier, synonyms, population supplementary concept word, anatomy supplementary concept word] | 64,990 |
| 27 | ((quality of life or QoL) adj10 (question$ or instrument or scale$1or score$1)).mp. [mp=title, book title, abstract, original title, name of substance word, subject heading word, floating sub-heading word, keyword heading word, organism supplementary concept word, protocol supplementary concept word, rare disease supplementary concept word, unique identifier, synonyms, population supplementary concept word, anatomy supplementary concept word] | 65,166 |
| 28 | or/24-27 | 496,979 |
| 29 | health state$.mp. | 8,926 |
| 30 | utilit$.mp. | 294,551 |
| 31 | Patient Preference/ or preference.mp. [mp=title, book title, abstract, original title, name of substance word, subject heading word, floating sub-heading word, keyword heading word, organism supplementary concept word, protocol supplementary concept word, rare disease supplementary concept word, unique identifier, synonyms, population supplementary concept word, anatomy supplementary concept word] | 147,028 |
| 32 | (map$ or regression).mp. [mp=title, book title, abstract, original title, name of substance word, subject heading word, floating sub-heading word, keyword heading word, organism supplementary concept word, protocol supplementary concept word, rare disease supplementary concept word, unique identifier, synonyms, population supplementary concept word, anatomy supplementary concept word] | 2,077,940 |
| 33 | health status.mp. or *Health Status/ | 188,434 |
| 34 | health status indicators.mp. or *Health Status Indicators/ | 24,520 |
| 35 | *"Activities of Daily Living"/ | 24,483 |
| 36 | *Health Surveys/ or health survey*.mp. | 106,945 |
| 37 | *Psychometrics/ or psychometric*.mp. | 122,270 |
| 38 | (health* year* equivalent* or HYE*).mp. [mp=title, book title, abstract, original title, name of substance word, subject heading word, floating sub-heading word, keyword heading word, organism supplementary concept word, protocol supplementary concept word, rare disease supplementary concept word, unique identifier, synonyms, population supplementary concept word, anatomy supplementary concept word] | 1,424 |
| 39 | or/29-38 | 2,814,377 |
| 40 | 28 and 39 | 118,120 |
| 41 | 23 or 40 [Ovid recomended QoL/Utility filter] | 165,160 |
| 42 | observational study/ or Case control study/ or Longitudinal study/ or Retrospective study/ or cross-sectional study/ or Cohort analysis/ or ((cohort or case control or follow up or observational or longitudinal or retrospective or cross sectional) adj (study or studies)).ti,ab. or register/ or registr*.ti,ab. or ((real adj3 world) or ((real-world or real world) adj2 (data or evidence or research))).ti,ab. [Clinical burden] | 3,298,381 |
| 43 | (cost minimi?ation analys* or (cost-minimi?ation adj1 analys*)).mp. [mp=title, book title, abstract, original title, name of substance word, subject heading word, floating sub-heading word, keyword heading word, organism supplementary concept word, protocol supplementary concept word, rare disease supplementary concept word, unique identifier, synonyms, population supplementary concept word, anatomy supplementary concept word] | 955 |
| 44 | exp Cost-Benefit Analysis/ | 96,078 |
| 45 | ((cost benefit adj1 analys*) or (cost-benefit adj1 analys*)).mp. [mp=title, book title, abstract, original title, name of substance word, subject heading word, floating sub-heading word, keyword heading word, organism supplementary concept word, protocol supplementary concept word, rare disease supplementary concept word, unique identifier, synonyms, population supplementary concept word, anatomy supplementary concept word] | 99,165 |
| 46 | (cost utility analys* or (cost-utility adj1 analys*)).mp. [mp=title, book title, abstract, original title, name of substance word, subject heading word, floating sub-heading word, keyword heading word, organism supplementary concept word, protocol supplementary concept word, rare disease supplementary concept word, unique identifier, synonyms, population supplementary concept word, anatomy supplementary concept word] | 4,438 |
| 47 | (cost consequence analys* or (cost-conseq* adj1 analys*)).mp. [mp=title, book title, abstract, original title, name of substance word, subject heading word, floating sub-heading word, keyword heading word, organism supplementary concept word, protocol supplementary concept word, rare disease supplementary concept word, unique identifier, synonyms, population supplementary concept word, anatomy supplementary concept word] | 424 |
| 48 | ((cost-effective* adj1 analys*) or "cost adj1 effectiveness adj1 analys*").mp. [mp=title, book title, abstract, original title, name of substance word, subject heading word, floating sub-heading word, keyword heading word, organism supplementary concept word, protocol supplementary concept word, rare disease supplementary concept word, unique identifier, synonyms, population supplementary concept word, anatomy supplementary concept word] | 18,178 |
| 49 | or/43-48 | 106,088 |
| 50 | ((economic or pharmacoeconomic) adj1 (evaluation or assessment or analys?s or stud*)).mp. [mp=title, book title, abstract, original title, name of substance word, subject heading word, floating sub-heading word, keyword heading word, organism supplementary concept word, protocol supplementary concept word, rare disease supplementary concept word, unique identifier, synonyms, population supplementary concept word, anatomy supplementary concept word] | 26,558 |
| 51 | ("CEA" or "CMA" or "CBA" or "CUA" or "CCA").mp. [mp=title, book title, abstract, original title, name of substance word, subject heading word, floating sub-heading word, keyword heading word, organism supplementary concept word, protocol supplementary concept word, rare disease supplementary concept word, unique identifier, synonyms, population supplementary concept word, anatomy supplementary concept word] | 75,808 |
| 52 | exp decision theory/ or exp decision trees/ | 13,908 |
| 53 | decision tree.mp. | 14,914 |
| 54 | models, economic/ | 11,360 |
| 55 | (markov or deterministic).mp. [mp=title, book title, abstract, original title, name of substance word, subject heading word, floating sub-heading word, keyword heading word, organism supplementary concept word, protocol supplementary concept word, rare disease supplementary concept word, unique identifier, synonyms, population supplementary concept word, anatomy supplementary concept word] | 55,908 |
| 56 | ((transition adj1 probabilit*) or (health adj1 stat*) or (sensitivity adj1 analys*) or (health adj1 outcome)).mp. [mp=title, book title, abstract, original title, name of substance word, subject heading word, floating sub-heading word, keyword heading word, organism supplementary concept word, protocol supplementary concept word, rare disease supplementary concept word, unique identifier, synonyms, population supplementary concept word, anatomy supplementary concept word] | 283,708 |
| 57 | ((patient level or patient-level or discrete event or discrete-event) adj1 simulat*).mp. [mp=title, book title, abstract, original title, name of substance word, subject heading word, floating sub-heading word, keyword heading word, organism supplementary concept word, protocol supplementary concept word, rare disease supplementary concept word, unique identifier, synonyms, population supplementary concept word, anatomy supplementary concept word] | 1,175 |
| 58 | (incremental-cost or incremental cost).mp. [mp=title, book title, abstract, original title, name of substance word, subject heading word, floating sub-heading word, keyword heading word, organism supplementary concept word, protocol supplementary concept word, rare disease supplementary concept word, unique identifier, synonyms, population supplementary concept word, anatomy supplementary concept word] | 16,874 |
| 59 | (ICER or QALY or DALY or WTP or TTO).mp. [mp=title, book title, abstract, original title, name of substance word, subject heading word, floating sub-heading word, keyword heading word, organism supplementary concept word, protocol supplementary concept word, rare disease supplementary concept word, unique identifier, synonyms, population supplementary concept word, anatomy supplementary concept word] | 22,265 |
| 60 | 50 and (or/51-59) | 9,671 |
| 61 | 49 or 60 [Ovid recomended economic evaluation filter] | 108,489 |
| 62 | 12 or 41 or 42 or 61 | 4,468,286 |
| 63 | 11 and 62 | 7,308 |
| 64 | exp Netherlands/ or exp Italy/ or exp Sweden/ or exp France/ or exp Spain/ or exp United Kingdom/ or (EU5 or europe or united kingdom or England or english or Scotland or scottish wales or welsh or Ireland or Irish or Spain or Spanish or france or french or italy or italian or Sweden or Swedish or netherlands or dutch).mp,cp,gc. | 14,605,017 |
| 65 | 63 and 64 | 3,240 |
| 66 | exp animal/ not (exp human/ and exp animal/) | 5,273,349 |
| 67 | exp note/ or exp editorial/ or exp letter/ or exp case report/ or exp Case study/ or exp Abstract report/ | 4,183,433 |
| 68 | 65 not (66 or 67) | 3,168 |
| 69 | limit 68 to yr="2015 -Current" | 1,940 |

Table S4: Evidence-Based Medicine Reviews - Cochrane Central Register of Controlled Trials (October 2024)

| **#** | **Search term** | **Results** |
| --- | --- | --- |
| 1 | sarcopenia.mp. or exp Sarcopenia/ | 2,760 |
| 2 | muscle atrophy.mp. or *Muscular Atrophy/ | 1,131 |
| 3 | frailty.mp. or exp Frailty/ | 4,065 |
| 4 | (Cancer or neoplasm or oncology or tumo?r or metasta$).mp. or exp Neoplasms/ | 292,310 |
| 5 | 1 or 2 or 3 or 4 | 298,743 |
| 6 | *Nutrition Therapy/ or exp malnutrition/ or *Nutritional Status/ or *Nutrition Assessment/ | 6,323 |
| 7 | (malnutrition or malnourish* or undernutrition or nutritional deficienc* or nutrition).ti. | 11,000 |
| 8 | (((diet* or nutri*) adj3 supplement*) or ((Liquid or drink or sip or sip feed or feed or formula) adj3 (supplement* or nutrition*)) or (protein$ adj2 supplement$)).tw. | 18,944 |
| 9 | ((high energy or high protein) adj5 supplement).mp. | 129 |
| 10 | (high energy high protein or hehp).mp. | 30 |
| 11 | 5 and (6 or 7 or 8 or 9 or 10) [Population of interest] | 4,363 |
| 12 | exp "cost of illness"/ or exp "Costs and Cost Analysis"/ or (cost analysis or cost-analysis).ti,ab,kw. or ((disease$ or sickness or illness) adj2 cost$).ti,ab,kw. or (economic adj5 (burden or cost$ or impact)).ti,ab,kw. or ((health care or healthcare or resource*) adj2 (use* or utili*)).ti,ab,kw. or ((direct or medical or indirect or nonmedical or societ* or employe*) adj2 (cost* or resource* or benefit*)).ti,ab,kw. or (cost$ or price$ or pricing).ti,ab,kw. or exp Drug Costs/ or exp Hospital Costs/ or Length of Stay/ or length of stay.ti,ab,kw. or exp Health Care Costs/ or ((fiscal or funding or financ$) adj2 (data or health care)).mp. or (Fees.mp. and Charges/) or Budgets/ or ((caregiver* or carer*) adj2 burden).ti,ab,kw. or exp *productivity/ or (productivity cost$ or productivity).ti,ab,kw. or (productivity loss or (productivity adj2 los*)).ti,ab,kw. or *Absenteeism/ or *Presenteeism/ or *Medical Leave/ or (absenteeism or presenteeism or sick leave).ti,ab,kw. or medication cost.mp. [Economic burden] | 150,828 |
| 13 | (EuroQOL 5-Dimension or Euroqol 5D or EQ-5D or EQ5D or Euroqol).mp. [mp=ti, ot, ab, fx, sh, hw, kw, tx, ct] | 16,862 |
| 14 | (Health utilities index or HUI).mp. [mp=ti, ot, ab, fx, sh, hw, kw, tx, ct] | 735 |
| 15 | (time trade off or time trade-off or ("TTO" adj2 "time trade")).mp. [mp=ti, ot, ab, fx, sh, hw, kw, tx, ct] | 567 |
| 16 | (short form 6D or short-form 6D).mp. [mp=ti, ot, ab, fx, sh, hw, kw, tx, ct] | 60 |
| 17 | (standard gamble or ("SG" adj2 "standard gamble")).mp. [mp=ti, ot, ab, fx, sh, hw, kw, tx, ct] | 298 |
| 18 | (15D or 16D or 17D).mp. [mp=ti, ot, ab, fx, sh, hw, kw, tx, ct] | 441 |
| 19 | (short form 36 or shortform 36 or SF-36 or SF36 or SF 36).mp. [mp=ti, ot, ab, fx, sh, hw, kw, tx, ct] | 21,167 |
| 20 | (short form 12 or shortform 12 or SF12 or SF-12 or SF 12).mp. [mp=ti, ot, ab, fx, sh, hw, kw, tx, ct] | 4,650 |
| 21 | (medical outcomes survey or MOS).mp. [mp=ti, ot, ab, fx, sh, hw, kw, tx, ct] | 3,514 |
| 22 | (Quality of wellbeing index or QWB).mp. [mp=ti, ot, ab, fx, sh, hw, kw, tx, ct] | 71 |
| 23 | or/13-22 | 43,864 |
| 24 | (QoL or HRQoL or HRQL).mp. [mp=ti, ot, ab, fx, sh, hw, kw, tx, ct] | 38,023 |
| 25 | quality of life.mp. or exp "Quality of Life"/ | 177,624 |
| 26 | (health related quality of life or health-related quality of life).mp. [mp=ti, ot, ab, fx, sh, hw, kw, tx, ct] | 27,296 |
| 27 | ((quality of life or QoL) adj10 (question$ or instrument or scale$1or score$1)).mp. [mp=ti, ot, ab, fx, sh, hw, kw, tx, ct] | 47,110 |
| 28 | or/24-27 | 186,022 |
| 29 | health state$.mp. | 3,256 |
| 30 | utilit$.mp. | 27,442 |
| 31 | Patient Preference/ or preference.mp. [mp=ti, ot, ab, fx, sh, hw, kw, tx, ct] | 16,014 |
| 32 | (map$ or regression).mp. [mp=ti, ot, ab, fx, sh, hw, kw, tx, ct] | 110,895 |
| 33 | health status.mp. or *Health Status/ | 205,92 |
| 34 | health status indicators.mp. or *Health Status Indicators/ | 1,344 |
| 35 | *"Activities of Daily Living"/ | 0 |
| 36 | *Health Surveys/ or health survey*.mp. | 10,362 |
| 37 | *Psychometrics/ or psychometric*.mp. | 7,670 |
| 38 | (health* year* equivalent* or HYE*).mp. [mp=ti, ot, ab, fx, sh, hw, kw, tx, ct] | 107 |
| 39 | or/29-38 | 181,933 |
| 40 | 28 and 39 | 33,401 |
| 41 | 23 or 40 [Ovid recomended QoL/Utility filter] | 64,935 |
| 42 | observational study/ or Case control study/ or Longitudinal study/ or Retrospective study/ or cross-sectional study/ or Cohort analysis/ or ((cohort or case control or follow up or observational or longitudinal or retrospective or cross sectional) adj (study or studies)).ti,ab. or register/ or registr*.ti,ab. or ((real adj3 world) or ((real-world or real world) adj2 (data or evidence or research))).ti,ab. [Clinical burden] | 210,687 |
| 43 | (cost minimi?ation analys* or (cost-minimi?ation adj1 analys*)).mp. [mp=ti, ot, ab, fx, sh, hw, kw, tx, ct] | 879 |
| 44 | exp Cost-Benefit Analysis/ | 23,837 |
| 45 | ((cost benefit adj1 analys*) or (cost-benefit adj1 analys*)).mp. [mp=ti, ot, ab, fx, sh, hw, kw, tx, ct] | 27,180 |
| 46 | (cost utility analys* or (cost-utility adj1 analys*)).mp. [mp=ti, ot, ab, fx, sh, hw, kw, tx, ct] | 5,518 |
| 47 | (cost consequence analys* or (cost-conseq* adj1 analys*)).mp. [mp=ti, ot, ab, fx, sh, hw, kw, tx, ct] | 1,867 |
| 48 | ((cost-effective* adj1 analys*) or "cost adj1 effectiveness adj1 analys*").mp. [mp=ti, ot, ab, fx, sh, hw, kw, tx, ct] | 24,378 |
| 49 | or/43-48 | 41,442 |
| 50 | ((economic or pharmacoeconomic) adj1 (evaluation or assessment or analys?s or stud*)).mp. [mp=ti, ot, ab, fx, sh, hw, kw, tx, ct] | 27,532 |
| 51 | ("CEA" or "CMA" or "CBA" or "CUA" or "CCA").mp. [mp=ti, ot, ab, fx, sh, hw, kw, tx, ct] | 4,263 |
| 52 | exp decision theory/ or exp decision trees/ | 1,095 |
| 53 | decision tree.mp. | 2,163 |
| 54 | models, economic/ | 1,828 |
| 55 | (markov or deterministic).mp. [mp=ti, ot, ab, fx, sh, hw, kw, tx, ct] | 6,270 |
| 56 | ((transition adj1 probabilit*) or (health adj1 stat*) or (sensitivity adj1 analys*) or (health adj1 outcome)).mp. [mp=ti, ot, ab, fx, sh, hw, kw, tx, ct] | 55,373 |
| 57 | ((patient level or patient-level or discrete event or discrete-event) adj1 simulat*).mp. [mp=ti, ot, ab, fx, sh, hw, kw, tx, ct] | 185 |
| 58 | (incremental-cost or incremental cost).mp. [mp=ti, ot, ab, fx, sh, hw, kw, tx, ct] | 10,442 |
| 59 | (ICER or QALY or DALY or WTP or TTO).mp. [mp=ti, ot, ab, fx, sh, hw, kw, tx, ct] | 9,389 |
| 60 | 50 and (or/51-59) | 14,755 |
| 61 | 49 or 60 [Ovid recomended economic evaluation filter] | 42,897 |
| 62 | 12 or 41 or 42 or 61 | 375,365 |
| 63 | 11 and 62 | 1,053 |
| 64 | exp Netherlands/ or exp Italy/ or exp Sweden/ or exp France/ or exp Spain/ or exp United Kingdom/ or (EU5 or europe or united kingdom or England or english or Scotland or scottish wales or welsh or Ireland or Irish or Spain or Spanish or france or french or italy or italian or Sweden or Swedish or netherlands or dutch).mp,cp,gc. | 431,532 |
| 65 | 63 and 64 | 306 |
| 66 | exp animal/ not (exp human/ and exp animal/) | 3,700 |
| 67 | exp note/ or exp editorial/ or exp letter/ or exp case report/ or exp Case study/ or exp Abstract report/ | 181 |
| 68 | 65 not (66 or 67) | 305 |
| 69 | limit 68 to yr="2015 -Current" | 251 |

Figure S1: PRISMA Flow Diagram

Abbreviations: CA, conference abstract; PRISMA, Preferred Reporting Items for Systematic reviews and Meta-Analyses.

## Indirect Treatment Comparison Calculations

Due to the limited availability of data for hospitalization rate and length of stay associated with standard oral nutritional supplements (S-ONS), alternative methods were used to estimate these values. Specifically, comparative efficacy of high-energy, high-protein oral nutritional supplements (HEHP ONS) vs S-ONS was derived via an indirect treatment comparison of relative risks for composite complications vs control, based on meta‑analysis data.^2^ Relative risks for complications were 0.82 for S-ONS vs control and 0.73 for HEHP ONS vs control, with an indirect treatment comparison (Bucher method) indicating that HEHP ONS was approximately 11% more effective than S-ONS in reducing complications (Figure S2). Hospitalization rate and length of stay data for S-ONS were subsequently derived by applying this proxy conversion factor to HEHP ONS data from Brown et al. (2020) (Table S5).^3^

Figure S2: Comparative Efficacy Calculations for HEHP ONS vs S-ONS

Abbreviations: H-HEHP, highest high energy, high protein, ONS, oral nutritional supplements; RR, relative risk; S-ONS, standard oral nutritional supplements.
* H-HEHP ONS assumed to be patients receiving ONS of ≥600 kcal/day; S-ONS assumed to be patients receiving ONS <600 kcal/ day.
† Cawood et al. (2023).^2^

Table S5: Complication Rates by Disease Population and ONS Treatment

| **Population** | **Complication type** | **Without ONS** | **S-ONS (calculated)** | **H-HEHP ONS** | **Reference** |
| --- | --- | --- | --- | --- | --- |
| Patients with frailty/sarcopenia and DRM | Hospital admission rate | 0.600 | 0.255 | 0.230 | Cawood et al. (2023)^2^  Brown et al. (2020)^3^ |
|  | Length of stay (days) | 6.770 | 2.458 | 2.220 | Cawood et al. (2023)^2^  Brown et al. (2020)^3^ |

Abbreviations: DRM, disease-related malnutrition; H-HEHP, highest high-energy, high-protein; ONS, oral nutritional supplements; S-ONS, standard oral nutritional supplements.
Note: Values represent rates/proportions or days as indicated by the complication type.
Note: Calculations were performed in the cost calculator using source values to all reported decimal places; reported values were rounded to three decimal places for presentation.

## Calculations for Per-Patient Analysis

The treatment and complication costs per patient were provided in Table 3. Treatment costs per patient were calculated by first estimating the number of bottles required per day. The number of bottles required per day was calculated by dividing the target energy intake threshold of 500 kcal/day by the energy content (kcal) of a single bottle (Table 2).

*Bottles required per day = target energy intake threshold of 500 kcal / energy content of one bottle*

The daily cost of nutrition for each product, defined as the product of the price per bottle (Table 2) and the number of bottles consumed per day (Table 2), was then calculated.

*Daily cost of nutrition = Price per bottle × number of bottles consumed per day*

This was multiplied by the average duration of use (Table 2) to estimate the per‑patient treatment cost.

*Per-patient treatment cost = Daily cost of nutrition × Average duration of use*

Where multiple products were used to determine the average cost per patient for a given nutrition type (e.g. S-ONS or H-HEHP ONS), costs were calculated using a weighted average approach based on current product utilization. Specifically, the per-patient treatment cost for each product (Table  2) was multiplied by its corresponding weight (average usage of product) (Table 2), the resulting values were summed, and the total was divided by the sum of the weights. This approach normalizes the weights and generates a weighted average cost per patient. The formula used to calculate the average cost per patient receiving H-HEHP ONS is provided below and can be adapted for S-ONS.

*Treatment cost (average) = (Per-patient treatment cost_[product A]_ × weight_[product A]_) + (Per-patient treatment cost_[product B]_ × weight_[product B]_) + (Per-patient treatment cost_[product C]_ × weight_[product C]_) / (Weight_[product A]_ + weight_[product B]_ + weight_[product C]_)*

Complication costs per patient were calculated as the product of the hospital admission rate (Table S5), the average length of hospital stay (Table S5), and the cost of hospital bed per day (Table S6).

*Complication cost per treatment = Hospital admission rate × Average length of hospital stay × Cost of hospital bed per day*

Total cost per patient was calculated as the sum of treatment and complication costs.

*Total cost per patient = Treatment costs + Complication costs*

Cost savings per patient associated with H-HEHP use were estimated as the difference in total cost between S-ONS and H­HEHP ONS treatments.

*Cost savings per patient due to H-HEHP use = Total cost of S-ONS treatment – Total cost of H-HEHP treatment*

## Calculations for Population-Level Analysis

The treatment and complication costs per population were provided in Table 4. The total number of patients with frailty/sarcopenia and DRM was 799,487. Of these, 40% (n=319,795) received ONS. Among patients receiving ONS, 73% (n=233,450) received H-HEHP ONS, while the remainder (n=86,345) received S-ONS.

*Treatment cost calculations*

Treatment costs per scenario (no ONS, S-ONS, and H-HEHP ONS) were calculated by multiplying the per‑patient treatment cost (Table 3) by the number of patients (Table S7).

*Treatment cost per scenario = Per-patient treatment cost associated with scenario × Number of patients per scenario*

Total treatment cost was calculated as the sum of treatment costs across all scenarios (no ONS, S­ONS, and H-HEHP ONS).

*Total treatment cost = Cost of no ONS treatment + Cost of S-ONS treatment + Cost of H-HEHP treatment*

*Complication cost calculations*

Complication costs per scenario (no ONS, S-ONS, and H-HEHP ONS) were calculated by multiplying the per‑patient complication cost by the number of patients.

*Complication cost per scenario = Scenario per-patient complication cost × Number of patients per scenario*

Cost savings associated with H-HEHP ONS were calculated as the difference in total costs between H­HEHP ONS and S-ONS.

*Cost savings per patient due to H-HEHP use = Total cost of S-ONS treatment – Total cost of H-HEHP treatment*

## Complication Costs

Table S6: Complication Costs for Patients with DRM (2024 Euros)

| **Cost item** | **Unit cost (€)** | **Source** |
| --- | --- | --- |
| Hospital bed day | 585.00 | ScanSante^4^ |

Abbreviation: DRM, disease-related malnutrition.

## Number of Community‑Dwelling Patients with Frailty/Sarcopenia and DRM across ONS Scenarios

Table S7: Number of Community‑Dwelling Patients with Frailty/Sarcopenia and DRM across ONS Scenarios (No ONS, S-ONS, and H-HEHP ONS)

| **Community dwelling patients with frailty/sarcopenia and DRM market scenario** | **Patients not receiving ONS** | **Patients receiving S-ONS** | **Patients receiving H-HEHP ONS** | **Total** |
| --- | --- | --- | --- | --- |
| Scenario 1: No ONS usage | 799,487 | – | – | 799,487 |
| Scenario 2: Only S­ONS available | 479,692 | 319,795 | – | 799,487 |
| Scenario 3: H-HEHP ONS available* | 479,692 | 86,345 | 233,450 | 799,487 |

Abbreviations: DRM, disease-related malnutrition; H-HEHP, highest high-energy, high-protein; ONS, oral nutritional supplements; S-ONS, standard oral nutritional supplements.
* Using current data on ONS usage and relative market shares of H-HEHP ONS vs S-ONS.
Note: Calculations were performed in the cost calculator using source values to all reported decimal places; reported values were rounded to the nearest whole number for presentation.

## Sensitivity Analysis

Figure S3: Sensitivity Analysis for Patients with Frailty/Sarcopenia and DRM

Abbreviations: DRM, disease-related malnutrition; H-HEHP, highest high-energy, high-protein; ONS, oral nutritional supplements.

## References

1.	Rives-Lange C, Desplas D, Zureik M. Ambulatory artificial nutrition support use in adolescents and adults: A nationwide population-based descriptive cohort study. JPEN J Parenter Enteral Nutr. 2026 Jan;50(1):67-76.

2.	Cawood AL, Burden ST, Smith T, Stratton RJ. A systematic review and meta-analysis of the effects of community use of oral nutritional supplements on clinical outcomes. Ageing Res Rev. 2023 Jul;88:101953.

3.	Brown F, Fry G, Cawood A, Stratton R. Economic Impact of Implementing Malnutrition Screening and Nutritional Management in Older Adults in General Practice. J Nutr Health Aging. 2020;24(3):305-11.

4.	ScanSante. Référentiel National de coût des unités d'oeuvres. Available at https://www.scansante.fr/applications/cout-dunites-doeuvre. Accessed 30 October 2025.
